# Evaluation of a Cost-Effective Novel Diagnostic Method for Lumbar Herniated Disc with Knee-Osteoarthritis: A Randomized Sample Study

**DOI:** 10.3390/medsci7060069

**Published:** 2019-06-12

**Authors:** Ganguly Apurba

**Affiliations:** Department of Research and Development, OPTM Research Institute, 145 Rashbehari Avenue, Kolkata 700029, India; apurbaganguly15@gmail.com; Tel.: +91-9830389616

**Keywords:** lumbar herniated disc, knee osteoarthritic, diagnostic protocol, biomarkers, outcome measures, lower extremity

## Abstract

The aim of this study was to determine a cost-effective diagnostic method for lumbar herniated disc with knee osteoarthritis (LHD-KOA) based on aberrant outcome measures, levels of biomarkers, and examination of the lower-extremity. Data were separately analyzed for each cohort suffering with LHD-KOA (*n* = 108; 59.82 ± 7.15 years) and without LHD-KOA (*n* = 108; 58.81 ± 7.61 years), and findings were confirmed with radiological images. The aberrant-leg-features (bilateral: knee gaps between the short head of biceps femoris and the surface of the bed, diameters of calves and thighs, angles of straight leg raising, knee-flexion and -extension in a supine position) and biochemical parameters (Interleukin-10, Tumor necrosis factor-alpha, C-reactive protein, creatine kinase-muscle, and Aldolase-A), and outcome measures, Western Ontario and McMaster Universities osteoarthritis index (WOMAC), knee-injury osteoarthritis outcomes scale (KOOS), Oswestry disability index (ODI), and body mass index (BMI)for participants with and without LHD-KOA were evaluated with appropriate techniques. All the subjects underwent standardized physical examination and completed a questionnaire. The risk ratios and mean ± standard deviations of biomarkers, anatomical features, and outcome measures of the experimental subjects were highly significant compared to controls (*p* < 0.0001). Results suggest that monitoring the studied aberrant outcome measures, biomarkers, and lower-anatomical features may be a cost-effective diagnostic tool for LHD-KOA. Further research is recommended for an alternative treatment protocol for LHD-KOA.

## 1. Introduction

In a previous study, the author established that there is a close relationship between degenerative changes in the lumbar region and bilateral knee osteoarthritis (KOA) [1]. The lumbar herniated disc (LHD) is the most common among different types of low back pain [2]. The slipped disc or lumbar herniated disc (LHD) usually occurs due to the compression of an existing nerve root, L_4_ or L_5_ at the L_4_-L_5_ or L_5_-S_1_ level of the spine [3,4,5,6]. According to Lorato et al., extracellular matrix remodeling, particularly of the elastic fiber system, vimentin immunopositive cells, oxytalan fibers, and apoplosis, are collaborated with herniated disc degenerative disease [7]. ALHD is characterized by a problem with lumbar intervertebral disc degeneration whereby the nucleus pulposus, a gel-like substance, protrudes through a crack of the outer-wall of the round spongy cartilage called the intervertebral disc, resulting in the compression of a nearby nerve root and/or cauda equina causing inflammation, pain, numbness, or weakness in the leg leading to abnormal quality of life [8]. Knee osteoarthritis is a painful knee joint degenerative disease characterized by muscle wasting, weakness, pain symptoms, inflammation, connective tissue damage, joint effusion, restricted movement of joints, and crepitus in the joints resulting in potential loss of hyaline cartilage and bone hypertrophy [9,10]. Knee osteoarthritisis the most common musculoskeletal degenerative joint disease associated with significant health and welfare costs around the world [11]. The characteristic features of KOA are joint space narrowing or subchondral sclerosis due to degenerative changes in the bones, cartilage, menisci, ligaments, and synovial tissue, leading to pain, immobility, and often disability [12,13]. According to Lawrence, it can occur at any age, but is most common for men between the ages 20 to 60 years [6]. The common symptoms of LHD and KOA are numbness, tingling, weakness in the muscles, and pain in the spine radiating to the buttocks, thighs, calves, feet (which is also termed lumbar radiculopathy during LHD [8,14]), pain and stiffness of the joints, and muscle weakness during KOA [15]. Therefore, the combination of LHD and KOA has serious risk factors for mobility limitation and can lead to impaired quality of life [8,9,14,15,16]. Ganguly has reported in detail the consequences of acute cases of LDH and KOA and also their risk factors as described in previous studies [8,14,15,16]

The primary diagnosis for LHD-KOA is based on the diagnostic guidelines of the North America Spine Society’s (NASS) evidence-based guidelines for multidisciplinary spine care and the Osteoarthritis Research Society International classification score establish guidelines for the diagnosis of KOA progression which includes patient’s history with physical examination, along with specific clinical outcomes, X-ray, magnetic resonance imaging (MRI) or computerized tomography (CT) scan, or discogram, or myelogram, or electromyography (EMG); and/or nerve conduction velocity (NCV) test [17,18,19,20,21,22,23,24,25].

The patient’s history with physical examination comprises current symptoms of pain (mild to severe), radiation or travel to other parts of the body, whether anything reduces the pain or makes it worse, range of motion of the lower limbs with the observation of neurological symptoms such as reflexes, identification of tender regions in the back and the legs, muscle strength, walking ability, and sensitivity to touch. The clinical outcome measure such as Western Ontario and McMaster Universities Osteoarthritis Index (WOMAC) is occasionally assessed. After the physical examination and outcome measures to confirm LHD-KOA, various other expensive and time-consuming diagnostic methods are applied to various regions of the body, such as X-rays of the lumbo-sacral spine and both the knee joints, which can reveal gross bony abnormalities such as fractures, compression between the vertebrae of spine and bones of knee joints, formation of osteophytes, or arthritis; magnetic resonance imaging (MRI) or computerized tomography (CT)scans of the spine and both knee joints that can pinpoint the damage condition of the soft tissues, ligament, osteophytes, and the affected nerves in the spine and the knee joints; a discogram that can be pinpoint the cracks in the individual disc; and finally a myelogram to check the herniated disc exerting any pressure on the spinal cord and nerves. Interestingly, it was found that LHD and pain on other joints may directly cause higher knee pain due to the biomechanical interrelationship of joints in the kinetic chain [26,27,28,29,30]. Ganguly [1], investigated that degenerative changes in the lumbar region always lead to bilateral degenerative changes in knee-joints and viceversa in which the sensation of pain cannot be the only parameter of degeneration. 

Major research works have been reported that low back pain LBP may lead to KOA [26,27,28,29,30] but nobody has studied the details of pain parameters based on various internationally-acclaimed outcome measures, biochemical effects on the serum of the patients, and anatomical features of the lower limbs during LHD associated with KOA. 

Therefore, in view of contractile regulation, function, and muscle metabolism, as suggested by Musumeci et al. [31], the present study has suggested an alternative diagnostic protocol for LHD-KOA with minimum cost and significant duration, even at the early stage of LHD-KOA, based on variabilities in: (1) clinical outcome measures including impaired quality of life along with obesity, (2) the biochemically-assessed status of inflammation, muscle degeneration, and skeletal muscle damage, and (3)lower anatomical features including muscle stiffness, wasting, atrophy, and restricted movements of joints. This approach is in response to the mysterious, costly, conventional diagnostic techniques mainly involving the study of diagnostic images [17,18,19,20,21,22,23,24,25] having several serious, unavoidable limitations [8,14]. Moreover, several researchers have emphasized that non-pharmacologic behavioral rehabilitation, treatment, and prevention in KOA, such as exercise and acupuncture, have many risk factors including obesity, muscles soreness, and joint tissue inflammation [32], and these benefits depend upon patient phenotypes [33].

The main common phenomena in LHD-KOA are pain and deteriorating psychometric quality of life which indicates the disease progression [29]. Suri et al. [26] examined the association between concurrent LHD and other musculoskeletal pain comorbidity with knee pain severity in symptomatic KOA. According to Wolfe [34], WOMAC function, pain, and stiffness score analyses can be suitable for low back pain, symptom counts, fatigue, and depression in osteoarthritis, rheumatoid arthritis, and fibromyalgia. Therefore, the appropriate pain parameters are suggested to be well-thought-out as per internationally approved clinical outcome measures such as WOMAC [35], knee-injury osteoarthritis outcomes scale (KOOS) [36], and Oswestry disability index (ODI) [37], along with obesity which is another major causing factor of pain and disability as assessed by body mass index (BMI) [38].

In order to identify the second-most common features in LHD-KOA namely, inflammation, connective tissue damage, skeletal muscle damage, and nerve functions, biochemical parameters such as Interleukin-10 (IL-10) [39], tumour necrosis factor-alpha (TNF-α) [40], C-reactive protein (CRP) [41,42], creatine kinase-muscle (CK-MM) [41,43,44], and Aldolase-A (AldoA) [41,45] have been proposed. Finally, the measurements of deranged lower-anatomical parameters have also recommended in connection with muscle stiffness, wasting, and atrophy (bulging) and range of motion of various joints such as the knee gap between the short head of biceps femoris and the surface of the bed in supine (KGB), diameter of calf muscles (DCM), diameter of thigh muscles (DTM), angle of straight-leg raising (SLR), knee flexion in supine (KFS), and knee extension in supine (KES).

The objective of present study was to attempt to diagnose the risk factors of LHD with KOA cost effectively even at early prognostic stage by analyzing the abnormal internationally-acclaimed functional disabilities such as WOMAC, KOOS, ODI, and BMI anomalous serum levels of biochemical parameters such as IL-10,TNF-α, CRP, CK-MM, and AldoA, and deranged lower-anatomical features (KGB, DCM, DTM, SLR, KFS, and KES) in combination with abnormal radiological images as assessed by the Kellgren–Lawrance (KL) grading scale of the experimental cohorts compared with the healthy control subjects.

Therefore, the present study presents novelty concepts for the diagnosis of LHD-KOA, into the categories of relevant internationally approved clinical outcome measures, specific biomarkers, and neuro-muscular lower leg anatomical features.

## 2. Materials and Methods

### 2.1. Study Design and Subjects

From eight centers of OPTM Health Care (P) Ltd, India, 315 cohorts, aged 45–79 years old, were recruited in the study between June 2017 to September 2018; based on the sign and symptoms as described in the previous studies [46].

The study protocol was approved by the OPTM Research Institute Ethics Committee. The institute is registered with the government of India under prescribed jurisdiction. An Institutional Review Board-approved consent form for the physical examinations, blood sample collections, and radiological images required for the study was signed by all participants in the first phase of the screening procedure.

After evaluating the exclusion criteria of 99 cohorts as mentioned in the previous studies [46,47], 108 (63 females and 45 males) of the remaining 216 subjects with significant pain syndromes, discomfort, imbalanced quality of life, impaired joint and limb functions due to inflammation, muscle wasting, weakness, and degeneration in multiple regions of the body, as evidenced by elevated levels of biomarkers (IL-10, TNF-α, CRP, CK-MM, and AldoA), and radiological images (CT-scan or X-ray or MRI) were considered as experimental subjects, and termed as subjects with LHD-KOA. The remaining 108 (63 females and 45 males) subjects who had no complain of pain, or no signs of LHD-KOA as evidenced by the analyses of studied biochemical markers and radiological images were considered as healthy control subjects and termed as subjects without LHD-KOA. Each subject completed a questionnaire at the baseline and summarized in Table 1.

### 2.2. Evaluation of Internationally-Approved Clinical Outcome Measures Including Body Mass Index

Observation of the patient’s perceived symptoms of pain intensity in the last 24 h for pain, stiffness, and functional disability of individual patient under WOMAC [35], KOOS [36] to assess the patient’s opinion about their knee and associated problems including quality of life, and ODI [37] for low back functional outcome to assess a patient’s permanent functional disability were evaluated separately for each cohort of experimental and control groups. The assessment of BMI [38] has been calculated individually for both the groups as per previous study [8].

### 2.3. Evaluation of Specific Biochemical Parameters in Blood

Collected blood samples were centrifuged at 1000× *g* for 10 min at 4 °C (Cryo Scientific Systems Pvt. Ltd., Chennai, Tamil Nadu, India) to obtain serum for each subject of experimental and control groups. The serums were used to analyze the biomarkers such as IL-10, TNF-α, CRP, CK-MM, and AldoA for each subject of both the groups separately. All the biomarkers were measured according to the methods and protocols elaborated in detail in the previous studies [8,41]. Each test for each patient has been rechecked by the BS-240 Mindray fully automated biochemistry analyzer before reporting the final test results for both the groups. 

### 2.4. Evaluation of Pearson’s Correlation Coefficients in Relations to Each Anatomical and Biochemical Parameter

To determine the predictive values of each deranged anatomical feature with each biochemical marker in patients with LHD-KOH, the Pearson’s correlation coefficients were evaluated between each anatomical parameter and biochemical marker (IL-10, TNF-α, CRP, CK-MM, and AldoA) along with their respective p-values. 

### 2.5. Evaluation of Anatomical Parameters

Physical examinations were evaluated for the measurements of the lower anatomical parameters such as KGB, DTM, DCM, SLR, KFS, and KES for each subject of both the groups. The measurements of the aforesaid anatomical parameters were elaborated in the previous studies [48].

### 2.6. Evaluation of Lumbar Spine and Knee Joints Radiographic Assessment under Kellgren–Lawrance Grading Scale

Lateral radiographs of the lumbar spine and anterior–posterior (AP) views of both knee-joints were obtained for all the cohorts of both the groups. Radiographs were classified and scored for lumbar degenerative intervertebral levels from L_1_-L_2_ to L_5_-S_1_ and osteoarthritic changes in knee-joints using Kellgren–Lawrance (KL) grading scale developed by Kellgren and Lawrence [49]

### 2.7. External Study Reviewers

All results and data of experimental and control groups separately were evaluated by an external reviewing panel, not in contract with the registry patients.

### 2.8. Data Collection and Statistical Analysis

Data were summarized using descriptive statistics for continuous variables (e.g., mean, standard deviation (SD), number of patients), frequency tables, risk ratios for discrete variables, and 95% confidence intervals. The mean, standard deviations, risk ratio, their 95% confidence intervals (CI), and *p*-values for all the outcome measures, biochemical, and lower-anatomical parameters were evaluated separately by gender for both the groups. Statistical analyses were done by using software (Graph Pad Prism, Version5.0, San Diego, CA, USA) with repeated measures for student-*t* test to determine significant values at *p* < 0.05 level along with r (Pearson’s correlation coefficient) values to determine strong and weak correlation among two variables for measuring different improvement parameters of combined sex, female, and male patients separately. An alpha level of 5% was established i.e., a *p*-value less than 0.05 was considered statistically significant.

## 3. Results

### 3.1. Enrolment and Baseline Characteristics of Patients

Two-hundred and sixteen subjects were included in the study and divided into equal numbers between the experimental group (with LHD-KOA) and control group (without LHD-KOA), fully described in Table 1.

### 3.2. Internationally-Approved Pain Related Outcome Measurements and Body Mass Index

**Figure 1:**
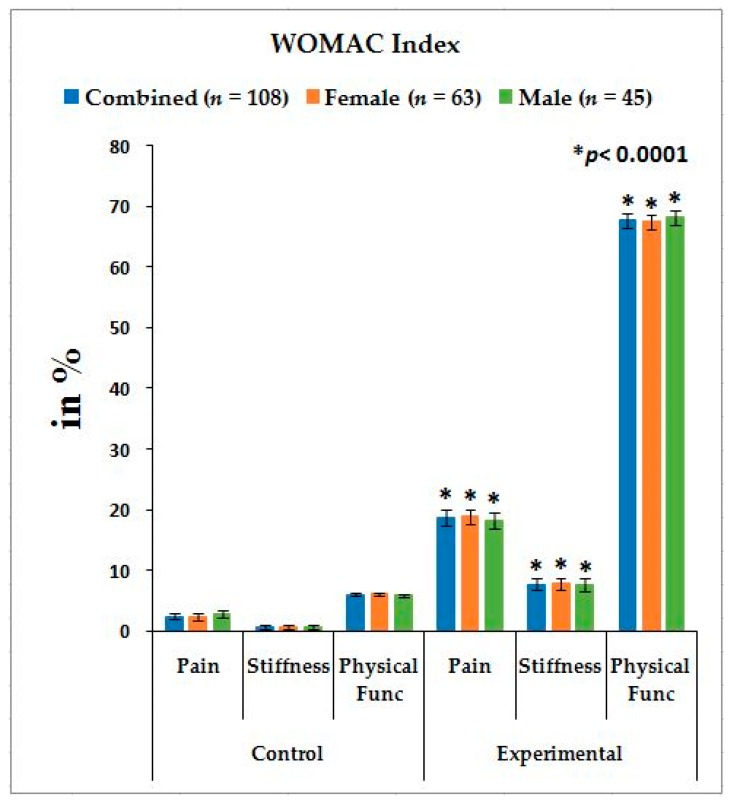
Graphical representation of international acclaimed pain outcome measurements under Western Ontario and McMaster Universities Osteoarthritis Index (WOMAC) Index in comparison with control and experimental groups and their *p*-values.

**Figure 2:**
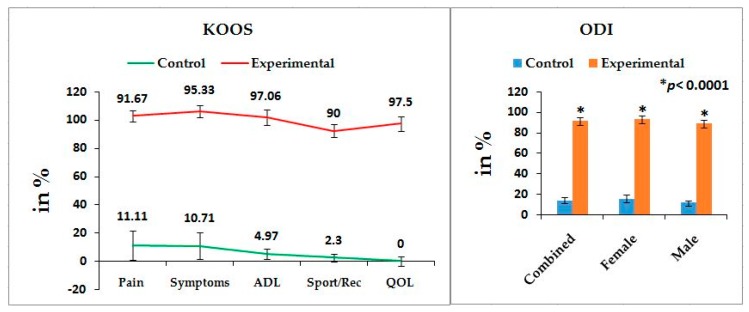
Graphical representation of international acclaimed pain outcome measurements under knee-injury osteoarthritis outcomes scale (KOOS) and Oswestry disability index (ODI) in comparison with control and experimental groups and their *p*-values.

**Figure 3:**
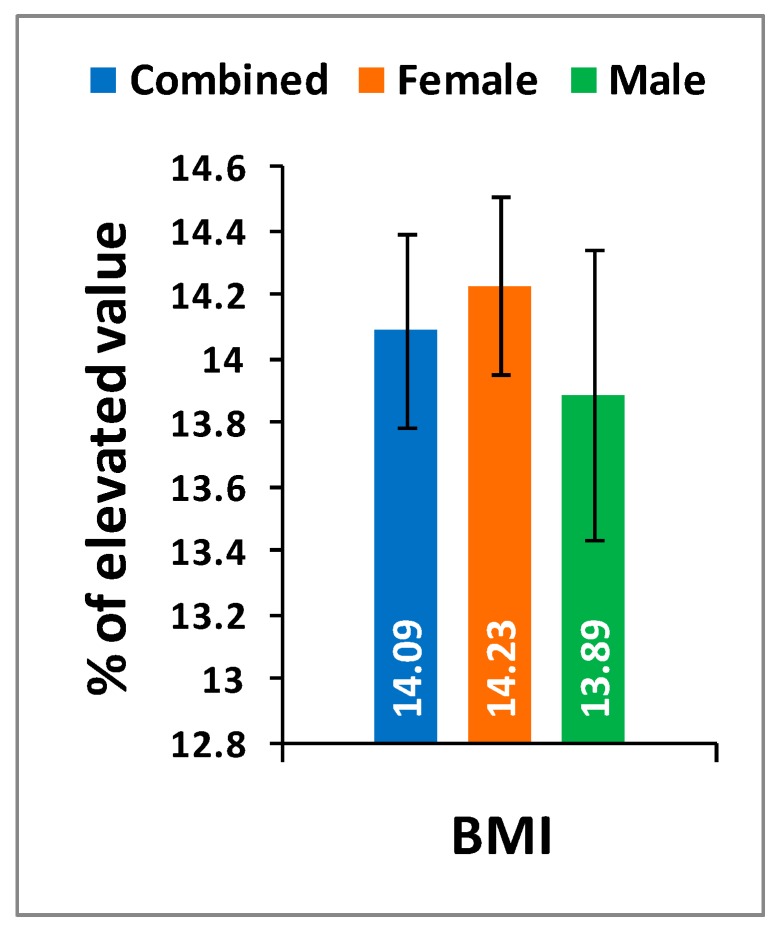
Percentage of graphical representation of body mass index (BMI) of experimental group in comparison with control group.

Table 2 shows the location of pain, sensory loss, and weakness in association with compression of nerve roots during lumbar herniated disc and the radiation of pain in the lower limbs associated with knee pain. The mean ± SD values: pain, stiffness, and physical functions under the WOMAC index; the five separately scored pain parameters under KOOS knee survey; pain-related parameters under ODI; and the percentage of increased obesity confirmed by BMI for combined-sex, female-only, and male-only patients of experimental groups were all highly significant (*p* < 0.0001), when compared with the subjects of control group (Figure 1, Figure 2 and Figure 3). 

### 3.3. Biochemical Parameters

**Figure 4:**
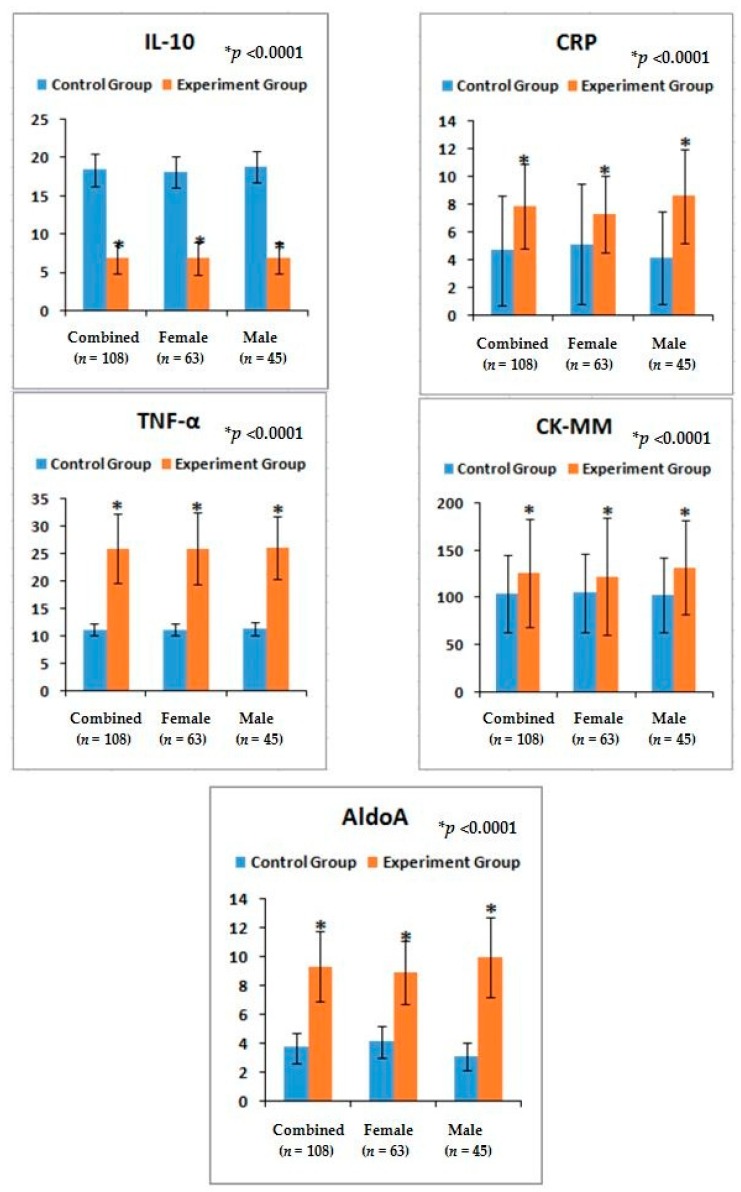
Graphical representation of biochemical parameters for IL-10, TNF-α, CRP, CK-MM, and AldoA in comparison with control and experimental groups and their *p*-values.

The risk ratios and their 95% CIs of all biochemical parameters such as IL-10, TNF-α, CRP, CK-MM, and AldoA of experimental subjects with their assessed cut off points (Table 3) were increased significantly (*p* < 0.0001) (Table 4) and the mean ± SD values of all biochemical parameters were also significant (*p* < 0.0001) (Figure 4) compared to control subjects (overall and separately by gender). Moreover, the predictive values of correlation coefficients of each lower anatomical feature with each level of biochemical marker in patients with LHD-KOA were highly significant (*p* < 0.0001) except CK-MM (Table 5).

### 3.4. Lower Anatomical Parameters

**Figure 5:**
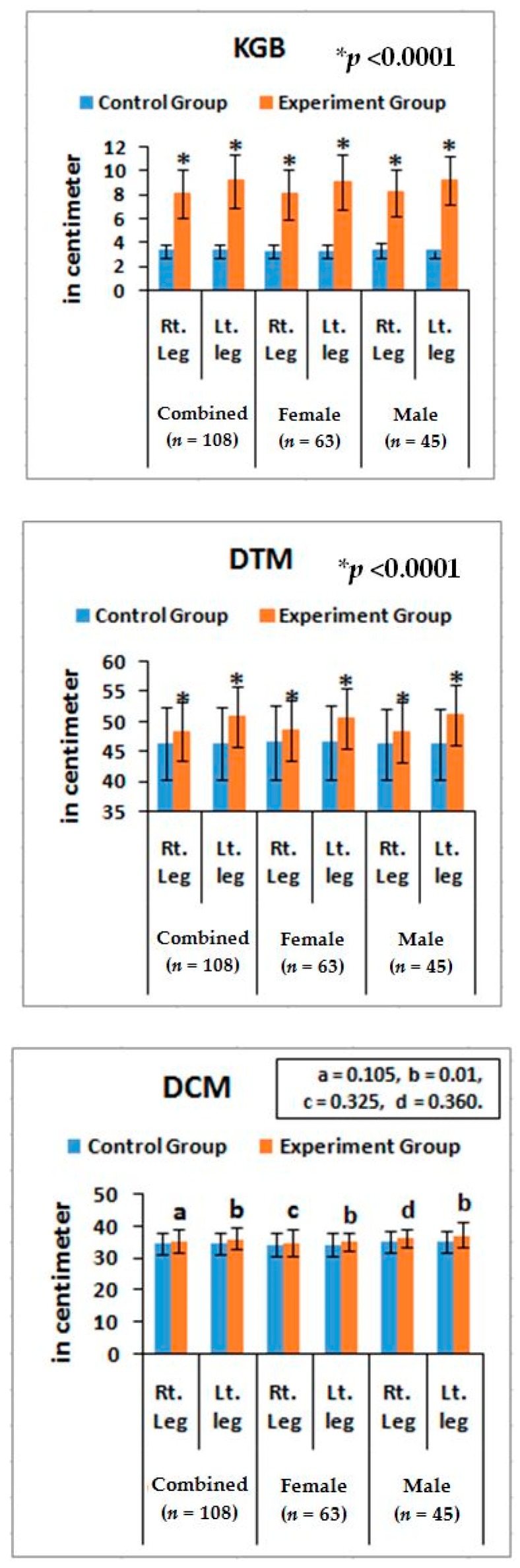
Graphical representation of anatomical features for KGB, DTM, and DCM in comparison with control and experimental groups and their *p*-values.

**Figure 6:**
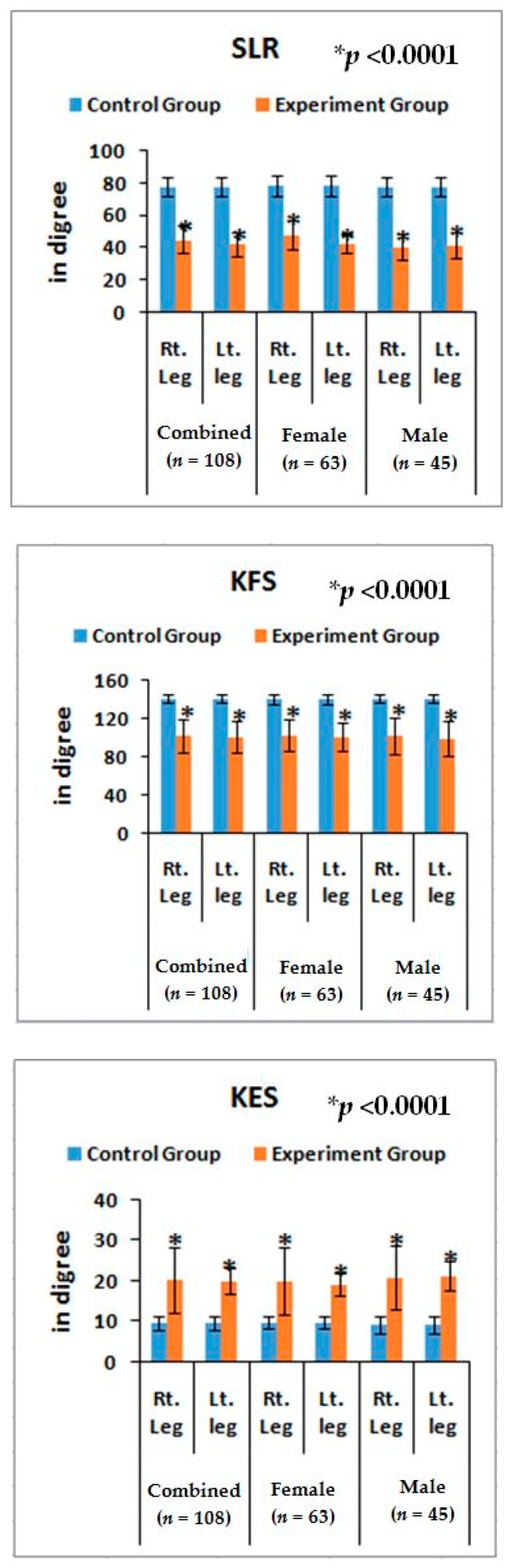
Graphical representation of anatomical features for angle of straight-leg raising (SLR), knee flexion in supine (KFS), and knee extension in supine (KES) in compression with control and experimental groups and their *p*-values.

The risk ratios and their 95% CIs of all the anatomical features of the lower limbs of experimental cohorts with their assessed cut off points (Table 3) were highly significant (*p* < 0.0001) when compared to the control cohorts for overall and separately by gender (Table 6). The mean ± SD values of all the anatomical features of the experimental cohorts were also highly significant (*p* < 0.0001) except the parameters of DTM and DCM and observed to be all asymmetrical for both the legs (Figure 5 and Figure 6). The correlation coefficients between the aberrant lower anatomical parameters and all the biochemical parameters were observed to be significant (*p* < 0.05) except DCM (right and left leg) (Table 5), when compared to the control cohorts.

### 3.5. Analysis of Radiological Images of Back Region and Knee Joints as Assessed by Kellgren–Lawrance Grading Scale

**Figure 7:**
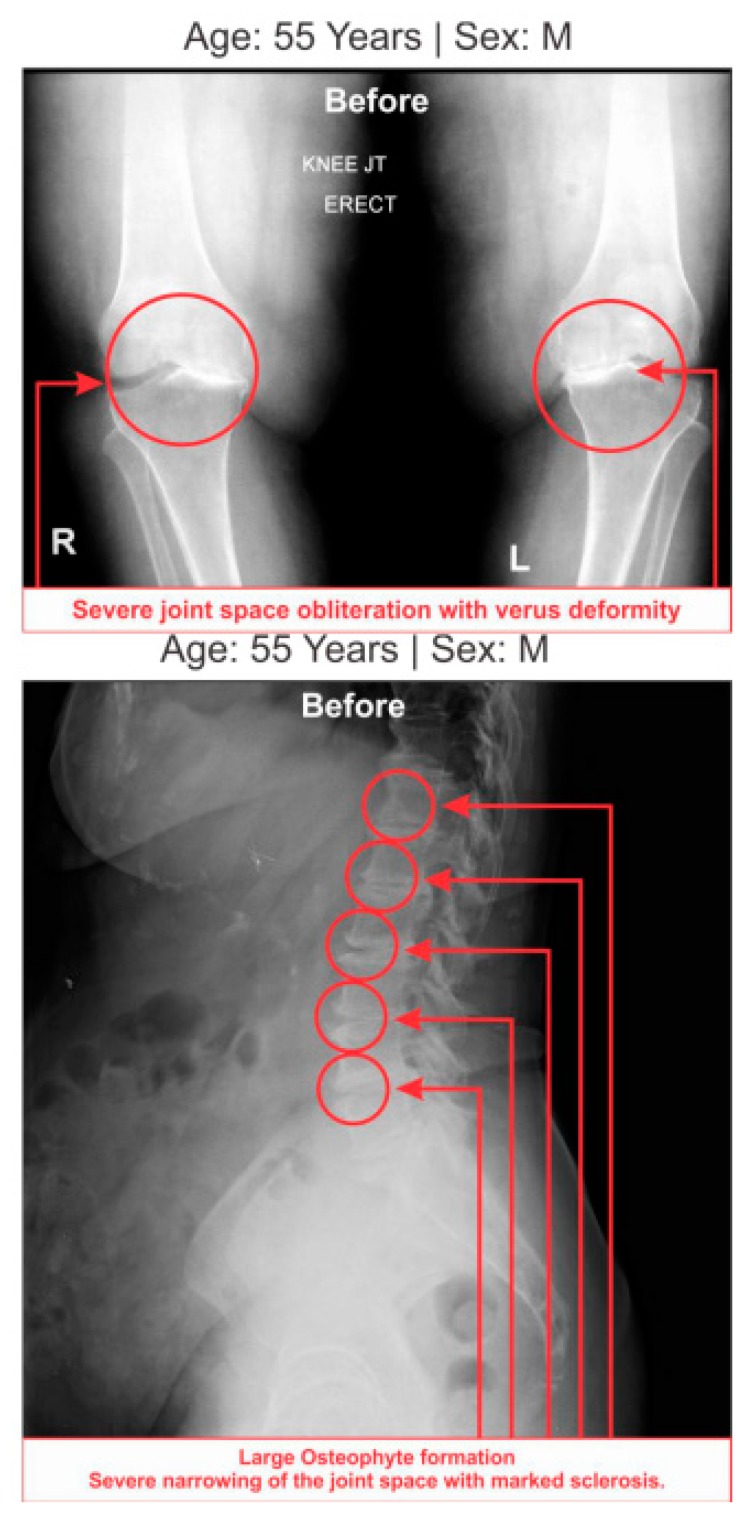
Radiological images of a patient suffering with lumbar herniated disc with knee-osteoarthritis (LHD-KOA).

All the anterior–posterior (AP) views of the knee joints and lateral views of lumbosacral spine X-ray reports of 108 patients with LHD-KOA exhibited degenerative changes, particularly in the medial tibio–femoral compartment, with marked joint space narrowing and bilateral varus/valgus deformities. Some cases exhibited near-complete medial compartment joint space obliteration and degenerative changes with osteophytes in the lumbar vertebrae. Table 7 shows the percentages of deterioration of grades under the KL grading scale for LHD and KOA. X-ray images of one such patient suffering with LHD associated with KOA are depicted in Figure 7.

## 4. Discussion

The results suggest that there is a close relationship between risk factors among internationally-approved pain-related clinical outcome measures including obesity, abnormal biochemical parameters such as IL-10, TNF-α, CRP, CK-MM, and Aldo A, deranged lower anatomical measurements, and KL grading scales to detect cost-effective diagnostic protocol for LHD-KOA in comparison with conventional costly diagnostic imaging such as X-ray, MRI, CT scan, or EMG. 

Magnetic resonance imaging is considered the gold standard for evaluating the relationship of disc material to soft tissue and neural structures during any musculoskeletal diseases. However, none of the conventional diagnostic tests focus on identifying the severity of pain, numbness, or weakness of the lower limbs, inflammatory status, muscle degeneration, and skeletal muscle damage affecting various joints in the lumbar region and knee. All of these methods are adopted in conventional diagnostic protocol in order to detect the degenerative changes occurring in the bony levels during acute stage of disorders, not in the early stage of disorder(s) or in the detection of damages at the levels of the connective tissues. Therefore, in the analyses of connective tissues, blood factors are the main important parameters for the detection of any diseases or disorders. Therefore, several researchers have suggested that there is need for an economical, accurate, and non-invasive diagnostic protocol for patients with acute musculoskeletal diseases instead of confirmed, expensive, advanced-diagnostic imaging such as MRI, EMG, CT-melograph, or painful nerve conduction tests [8,14,15,24,25,26]. Moreover, the author has previously elaborated in detail the persistence, advantages, and limitations of costly diagnostic imaging for the diagnosis of LHD and KOA on various regions of the body [8,14,15,29,30].

Pain syndromes, obesity, and impaired quality of life are the major perceptive factors among patients in any musculoskeletal disorders specially LHD-KOA [8,14,15,48]. Wolfe [34] and Suri et al. [26] have suggested that the analyses of pain, stiffness, and physical functions under the WOMAC scale among patients with KOA are influenced by low back pain which is the main factor of LHD. But the author suggests that the analysis of KOOS is more accurate than WOMAC in cases of patients suffering with LHD-KOA. KOOS was developed by Roos et al. [36] from 42 self-administered questionnaires based on the study of 24 self-administered questionnaires from the WOMAC Osteoarthritis Index developed in 1982 at Western Ontario and McMaster Universities [35] and both can be used on patients aged 13–79 years. It is interesting to note that the last two questions of the symptoms-domain referring to joint stiffness, the last five questions of the pain assessment domain referring to pain, and all 17 questions for the analysis of ability to move around or look after oneself under function of daily living in KOOS are exactly similar to WOMAC index. Therefore, the pain, stiffness of muscles, aberrant functional activities of daily living, and quality of life can be analyzed in more detail with the help of KOOS than with WOMAC during LHD-KOA. Table 2 shows the location of pain, sensory loss, and weakness in the lower extremities in association with the compression of nerve roots during LHD. In the present study, it is observed that all the internationally-acclaimed outcome measures such as WOMAC, KOOS, ODI, and BMI(as body weight is the important factor for any musculoskeletal disease) are increasingly significant during LHD-KOA compared to controls (Figure 1, Figure 2 and Figure 3) which supports the previous research [26,30,31,32,33,34,35,36,37,38].

Again, LHD and KOA are soft-tissue inflammatory diseases [49]. The author has already elaborated the reason for choosing two cytokines (TNF-α, a pro-inflammatory cytokine and IL-10, an anti-inflammatory) for the diagnosis of LHD-KOA in the previous study [8,14]. The present results indicate the phenomena for the increase ofIL-10serum levels, the decreasing trend for TNF-α, the serum level of CRP found to be elevated during inflammation in tissues from the standard value (<6 mg/L) [46,47], the CK-MM level elevated from the standard value (<168 U/L) in response to muscular dystrophy, connective tissue damage, [8,9,14,50,51,52,53], and AldoA level increases from the standard value of <7.6 U/L due to skeletal muscle damages, polymyositis, dermatomyositis, muscular dystrophy, and inflammatory muscle disease and bone erosion [53] during LHD-KOA. The result is there is restricted movement of the joints with stiffness and decreased range of motion which firmly indicate the research criteria. There are no earlier studies on the relative risk ratio in combined affect with the serum levels of aforesaid biomarkers along with the alteration of anatomical features in LHD-KOA (Table 4 and Figure 4).

The present findings indicate predictive risk factors through the analysis of correlation coefficients between the risk factors of biochemical markers of inflammation, connective tissue damage, muscle degeneration, and skeletal muscle damage measured with the help of analyses of serum IL-10, TNF-α, CRP, CK-MM, and AldoA and the aberrant lower extremities such as KGB, DCM, DTM, SLR, KFS, and KES of experimental cohorts. There findings were highly significant except in the case of CK-MM wherein the relation between the status of inflammation and damage of skeletal muscles is firmly established during LHD-KOA.

Although the researchers have analyzed the various movements of the lower limbs, range of motion of knee joints, visual inflammation/swelling, neurological deficits such as muscle strength, tendon reflexes, and sensory impairments during physical examination, the assessment of their diagnostic accuracy for LHD-KOA is ambiguous [54]. 

Furthermore, noticeable abnormalities are observed in the muscle strength, movement of joints, and muscle morphology during physical examination for LHD with KOA. The functionality of the lower back and knee joints is based on the complex interplay of different motion segments and muscular activities. The ability of the waist and legs to move in a normal range depends on the health of muscles, ligaments, bones, and individual joints. The results from the deranged lower-anatomical parameters indicate that there are substantial increasing or decreasing phenomena of the group of muscles connected with the spinal vertebrae and the legs, which were asymmetrical in respect to the measurements of KGB, DCM, DTM, SLR, KFS, and KES of the experimental cohorts indicating the muscular wasting, muscle weakness, and degeneration during LHD-KOA (Table 6 and Figure 5 and Figure 6). Table 7 shows the high-graded abnormal bony morphology both in the lumbar region (4.63% in grade 3 and 95.37% in grade 4) and knee joints (3.37% and 1.85% in grade 3 and 96.30% and 98.15% in grade 4 for right and left knee joints respectively) as assessed by the KL grading scale during LHD-KOA. This phenomenon also which confirmed with radiological images of one of the patients suffering with LHD-KOA as shown in (Figure 7).

In the present study, based on the World Health Organization’s International Classification of Functioning, Disability of Health [55], multidimensional approaches such as body function (pain and mobility in the joints), psychological factors (learned helplessness, mood, and pain copying), self-efficacy to modify personal factors, and body structures (cartilage, bone, and soft tissues), along with inflammatory status, muscle degeneration, skeletal muscle damage, and bone erosion as assessed by biochemical parameters, have been taken care of with the help of three parametric concepts of diagnostic protocol. These concepts are: internationally-acclaimed outcome measures (WOMAC, KOOS, ODI, and BMI), abnormal studied biochemical parameters, and deranged anatomical parameters. They may assist in the confirmation of degenerative changes both lumbar region and knee joints at an early stage with minimum cost [56,57,58]. It is interesting to note that Ganguly in 2019 has established a novel topical phytotherapy for normalization of aberrant leg-anatomical and -biochemical risks in failed spine surgery for LHD associated with knee-osteoarthritis [59]. The results of this study draw attention that the said trio-parametric conceptual diagnostic protocol as a suitable prognostic tool in its first-time evaluation to identify abnormality in both the lumbar region and knee-joints. Moreover, the present study contradicts the earlier study that women are more likely to have the problem than men, which may be the reason of small sample size of survey [6].

However, there are some limitations in this study. Firstly, we have a small sample size and have not yet confirmed whether these results are biased. Secondly, this study was restricted to patients who were not suffering with rheumatic diseases; osteochondritis diseases; congenital dysplasia; radicular syndrome; joint symptoms caused by malignant tumors; dermatomyositis and polymyositis diseases; iliopectineal or trochanteric bursitis; ischemic bone necrosis; bone and joint infectious diseases; cuts, wounds or any type of chronic skin and infectious diseases; parallel multiple drug dependence for concomitant diseases or risk conditions requiring drug treatment including psychiatric diseases; a history of cancer, including caranomatosis and granulocytic leukemia; a history of severe neurological diseases; a history of chronic liver, kidney, and heart diseases; and patients who did not agree to give blood sample, which may have been due to drug/alcohol addiction or pregnancy.

## 5. Conclusions

It is concluded that monitoring the risk ratio of biomarkers (IL-10, TNF-α, CRP, CK-MM, and AldoA) (Table 4 and Figure 4) and leg anatomical parameter (KGB, DTM, DCM, SLR, KFS, and KES) (Table 5 and Table 6 and Figure 5 and Figure 6) along with internationally-acclaimed functional disability outcome parameters (WOMAC Index, KOOS, ODI, and BMI) (Figure 1, Figure 2 and Figure 3), confirming findings with spine and knee joint radiographic images as assessed by the KL grading scales (Table 7 and Figure 7), may be a novel diagnostic protocol for detecting LHD-KOA with minimal cost and time. The results of the present work encourage further research on a cost-effective, non-invasive treatment procedure for LHD-KOA.

## Figures and Tables

**Table 1:** Demographic data and baseline characteristics of subjects.

Characteristics	Experimental Group	Control Group
No of subjects	108	108
Females	63 (58.33%)	63 (58.33%)
Age (yr), (mean (SD))	59.82 (7.15)	58.81(7.61)
Height (m), (SD)	1.55 (0.72)	1.51 (0.77)
Weight (kg.), (mean (SD))	76.24 (4.16)	62.43 (4.79)
BMI (kg/m^2^) (mean (SD))	31.77 (3.32)	27.75(3.39)
Period of suffering (yrs), (mean (SD))	5.74 (1.82)	-
**Indian ethnic group (%)**		
Bengali	28 (24.79)	29 (23.08)
Gujrati	9 (9.40)	12 (10.11)
Marwaree	10 (8.55)	11 (11.11)
Marathi	14 (13.67)	13 (12.82)
Tamil	12 (12.81)	12 (12.82)
Punjabi	14 (11.11)	10 (10.26)
Shindhi	10 (10.25)	12 (11.09)
North East India	11 (9.40)	9 (8.45)
**Food habits (%)**		
Vegetarian	75 (69.44)	69 (63.89)
Non-vegetarian	33 (30.56)	39 (36.11)
**Other habits (%)**		
Drinking excessive tea and coffee	22 (20.37)	12 (11.11)
Smoking	28 (25.93)	23 (21.30)
Drinking alcohol	21 (19.44)	13 (12.03)
Chewing tobacco	7 (6.48)	8 (7.41)
Free from other habits	30 (27.78)	52 (48.15)
**Work status (%)**		
Employed fulltime	30 (27.78)	29 (26.85)
Employed part time	11 (10.18)	10 (9.26)
Housewife/homemaker	22 (20.37)	23 (21.30)
Retired	19 (17.59)	21 (19.44)
Self employed	26 (24.08)	25 (23.15)
**Multiple complaints (%)**		
Constipation	64 (59.26)	22 (20.37)
Acidity andreflux	67 (62.04)	18 (16.67)
Insomnia	68 (62.96)	19 (17.59)
Varicose vein	39 (36.11)	15 (13.89)
Urinary incontinence	58 (53.70)	18 (16.67)
Crepitus during knee flexion	33 (30.55)	-
Morning stiffness (<30 min)	37 (34.26)	-
**Measures taken to diminish pain (%)**		
Using a lumbar belt	34 (31.48)	-
Using knee cap	55 (50.93)	
Using a sick	24 (22.22)	-
Using walker	18 (16.67)	-

SD: standard deviation; BMI: body mass index; yr(s): year(s).

**Table 2:** Location of pain, sensory loss, and weakness in association with compression of nerve roots during lumbar herniated disc.

Nerve	Location of Pain	Sensory Loss on	Weakness on
**L1**	Inguinal and medial thigh	Inguinal region	Flexion weakness is rare
**L2, L3–L4**	Back pain radiating into the anterior and medial aspect of upper thigh and medial lower leg.	Anterior thigh and sometimes medial lower leg	Hip flexion and adduction, knee extension and quadriceps. Diminished patella reflex.
**L5**	Back, radiating into buttock, lateral thigh, lateral calf and dorsum foot, great toe	Lateral calf, dorsum of the foot, web space between first and second toe.	Hip adduction, knee flexion and semitendinosus/ semimembranosus reflex.
**S1**	Back, radiating into buttock, lateral or posterior thigh, posterior calf, lateral plantar foot.	Posterior calf, lateral or planter aspect of foot.	Hip extension, knee flexion, plantar flexion of the foot; Achillestend on; Medial buttock, perineal and perianal region; Occasional urinary, fecal incontinence, and sexual dysfunction
**S2–S4**	Sacrum or buttock radiating into the posterior aspect of the leg or the perineum.	Medial buttock, perineal and perianal region	Absent bulbocavernosus and wink reflex

Source: Ganguly A, Ganguly D. Aberrant biomarkers, leg anatomy and pain parameters are the risk factors in lumbar-herniated disc: A novel diagnostic protocol. Journal of Orthopedics and Rheumatology 2018a; 5(2), 11.

**Table 3:** Analysis of cut off points of anatomical and biochemical parameters for the calculation of relative risk ratio.

Anatomical Parameter	Cut off Point	Biochemical Parameter	Cut off Point
KGB	≤4 cm	IL-10	>12 pg/mL
DTM	≤47 cm	TNF-α	<15 pg/mL
DCM	≤30 cm		
SLR	≥70 degree	CRP	<6 mg/L
KFS	≥138 degree	CK-MM	<168 U/L
KES	≤10 degree	AldoA	<7.60 U/L

KGB: knee gap between the short head of biceps femoris and the surface of the bed in supine; DTM: diameter of thigh muscles; DCM: diameter of calf muscles; SLR: angle of straight leg raising; KFS: knee flexion in supine; KES: knee extension in supine; IL-10: Interleukin-10; TNF-α: Tumor necrosis factor-alpha; CRP: C-reactive protein; CK-MM: Creatine kinase-muscle; AldoA: Aldolase-A.

**Table 4:** Analysis of risk ratios of biochemical parameters for combined-sex, female-only, and male-only.

Combined Sex (*n* = 108)				Female-Only (*n* = 63)			Male-Only (*n* = 45)		
	Risk Ratio	95% CI	*p*-Value	Risk Ratio	95% CI	*p*-Value	Risk Ratio	95% CI	*p*-Value
**IL-10**	4.00	2.52, 6.33	<0.0001	5.11	2.71, 9.65	<0.0001	2.75	1.41, 5.34	0.0028
**TNF-α**	21.20	9.00, 49.92	<0.0001	35.50	9.05, 139.26	<0.0001	8.75	3.47, 22.08	<0.0001
**CRP**	2.44	1.71, 3.50	<0.0001	2.65	1.68, 4.16	<0.0001	2.10	1.16, 3.81	<0.0001
**CK-MM**	11.87	2.07, 23.22	<0.0001	10.67	4.94, 23.05	<0.0001	15.50	4.00, 59.98	0.0001
**AldoA**	3.95	2.62, 5.96	<0.0001	4.17	2.43, 7.14	<0.0001	3.62	1.93, 6.82	0.0001

**Table 5:** Analysis of correlation coefficient and their *p*-values in relation to anatomical and biochemical parameters of experimental subjects.

Anatomical Parameter	Correlation Coefficient and Their *p*-Values	Biochemical Parameter				
		CRP	CK-MM	AldoA	TNF-α	IL-10
KGB (R)	Correlation coefficient	0.218	0.162	0.140	0.704	−0.676
	*p*-value	0.001	0.017	0.039	0.000	0.000
KGB (L)	Correlation coefficient	0.232	0.147	0.123	0.702	−0.678
	*p*-value	0.001	0.031	0.070	0.000	0.000
DTM (R)	Correlation coefficient	0.141	0.019	−0.049	0.119	−0.131
	*p*-value	0.038	0.777	0.477	0.081	0.055
DTM (L)	Correlation coefficient	0.147	0.031	−0.030	0.112	−0.128
	*p*-value	0.031	0.656	0.659	0.102	0.061
DCM (R)	Correlation coefficient	0.016	−0.042	−0.057	0.007	−0.003
	*p*-value	0.818	0.542	0.408	0.920	0.967
DCM (L)	Correlation coefficient	0.041	−0.016	−0.053	0.020	−0.021
	*p*-value	0.548	0.812	0.440	0.774	0.763
SLR (R)	Correlation coefficient	−0.241	−0.137	−0.165	−0.754	0.713
	*p*-value	0.000	0.044	0.015	0.000	0.000
SLR (L)	Correlation coefficient	−0.260	−0.130	−0.135	−0.765	0.731
	*p*-value	0.000	0.056	0.047	0.000	0.000
KFS (R)	Correlation coefficient	−0.255	−0.159	−0.147	−0.716	0.680
	*p*-value	0.000	0.019	0.031	0.000	0.000
KFS (L)	Correlation coefficient	−0.276	−0.124	−0.139	−0.725	0.694
	*p*-value	0.000	0.069	0.041	0.000	0.000
KES (R)	Correlation coefficient	0.228	0.142	0.157	0.719	−0.681
	*p*-value	0.001	0.037	0.021	0.000	0.000
KES (L)	Correlation coefficient	0.240	0.119	0.132	0.730	−0.692
	*p*-value	0.000	0.080	0.052	0.000	0.000

KGB: Knee gap between the short head of biceps femoris and the surface of the bed in supine; DTM: diameter of thigh muscles; DCM: diameter of calf muscles; SLR: angle of straight leg raising; KFS: knee flexion in supine; KES: knee extension in supine; (R): right; (L): left.

**Table 6:** Analysis of risk ratios of anatomical parameters for combined-sex, female only, and male only.

		Combined Sex (*n* = 108)			Female-Only (*n* = 63)			Male-Only (*n* = 45)		
		Risk Ratio	95% CI	*p*-Value	Risk Ratio	95% CI	*p*-Value	Risk Ratio	95% CI	*p*-Value
**KGB**	Right leg	2.52	1.96, 3.24	<0.0001	2.43	1.81, 3.26	<0.0001	2.75	1.71, 4.41	<0.0001
	Left leg	2.38	1.87, 3.03	<0.0001	2.52	1.86, 3.41	<0.0001	2.13	1.42, 3.19	0.0002
**DTM**	Right leg	1.26	0.99, 1.61	0.0579	1.02	0.77, 1.35	0.8661	1.78	1.12, 2.84	0.0142
	Left leg	1.30	1.02, 1.65	0.0304	1.10	0.83, 1.46	0.5017	1.86	1.18, 2.93	0.0079
**DCM**	Right leg	1.88	1.51, 2.33	<0.0001	1.69	1.32, 2.18	<0.0001	2.36	1.55, 3.59	0.0001
	Left leg	1.96	1.58, 2.43	<0.0001	1.72	1.34, 2.21	<0.0001	2.61	1.68, 4.07	<0.0001
**SLR**	Right leg	8.75	5.13, 14.93	<0.0001	7.36	4.50, 12.01	<0.0001	17.50	4.54, 67.37	<0.0001
	Left leg	7.36	4.50, 12.01	<0.0001	5.83	3.47, 9.79	<0.0001	16.50	4.27, 63.68	<0.0001
**KFS**	Right leg	2.60	2.03, 3.33	<0.0001	2.30	1.74, 3.03	<0.0001	3.50	2.06, 5.94	<0.0001
	Left leg	2.76	2.11, 3.59	<0.0001	2.43	1.81, 3.26	<0.0001	3.78	2.13, 6.69	<0.0001
**KES**	Right leg	2.54	1.99, 3.24	<0.0001	2.80	2.03, 3.85	<0.0001	2.12	1.46, 3.09	0.0001
	Left leg	2.76	2.13, 3.58	<0.0001	2.96	2.13, 4.11	<0.0001	2.43	1.60, 3.68	<0.0001

KGB: Knee gap between the short head of biceps femoris and the surface of the bed in supine; DTM: diameter of thigh muscles; DCM: diameter of calf muscles; SLR: angle of straight leg raising; KFS: knee flexion in supine; KES: knee extension in supine; CI: interval of confidence.

**Table 7:** Kellgren–Lawrance (KL) Grading scales for lumbar herniated disc (LHD) and knee-osteoarthritis (KOA) of experimental and control groups.

	Experimental Group		Control Group	
	No of Patient	%	No of Patient	%
**LHD**				
Grade 1:	None	None	101	93.52
Grade 2:	None	None	7	6.48
Grade 3:	5	4.63	-	-
Grade 4:	103	95.37	-	-
**KOA (Right knee)**				
Grade 1:	None	None	99	91.67
Grade 2:	None	None	9	8.33
Grade 3:	4	3.70	-	-
Grade 4:	104	96.30	-	-
**KOA (Left knee)**				
Grade 1:	None	None	102	94.44
Grade 2:	None	None	6	5.56
Grade 3:	2	1.85	-	-
Grade 4:	106	98.15	-	-

LHD: lumbar herniated disc; KOA: knee-osteoarthritis.
